# Penile Livedoid Vasculopathy: First Reported Case

**DOI:** 10.1155/2023/6920383

**Published:** 2023-07-04

**Authors:** Ahmad Hallak, William Bennett, Mohammed Adib Tanbir, Supriya R. Donthamsetty, Bethaney Vincent

**Affiliations:** ^1^Ochsner Medical Center, New Orleans, Louisiana, USA; ^2^Texas Tech University Health Sciences Center, USA

## Abstract

Livedoid vasculopathy is a thromboocclusive cutaneous vasculopathy manifested by livedoid changes, atrophie blanche, and ulceration. The pathogenesis is speculated to involve increasing coagulation or impaired thrombolysis leading to the occlusion of dermal blood vessels with fibrin thrombi. Livedoid vasculopathy is known to primarily affect the lower extremities. We report the first case of livedoid vasculopathy affecting the penis. A 60-year-old male was evaluated for a split urine stream with associated irritation and peeling of the skin of the glans penis. His penile ulcer continued to enlarge despite steroids and antibiotics. Due to diagnostic uncertainty, a biopsy was performed which revealed hyaline thrombi within the lumens of small vessels within the upper to mid dermis, fibrinoid material in the walls of these blood vessels and within the perivascular stroma with overlying and adjacent epidermal spongiosis, and mild perivascular lymphocytic infiltrate with a few scattered neutrophils most consistent with livedoid vasculitis. He was started on aspirin and pentoxifylline with limited improvement and was later started on apixaban with near-complete resolution in 6 months. Penile livedoid vasculopathy has not been previously reported in the English literature. Early diagnosis and treatment are imperative to limit morbidity.

## 1. Introduction

Livedoid vasculopathy is a thromboocclusive cutaneous vasculopathy manifested by livedoid changes, atrophie blanche, and ulceration. It was first described in 1955 [1]. Although poorly understood, the pathogenesis is speculated to involve increasing coagulation or impaired thrombolysis leading to occlusion of dermal venules with fibrin thrombi in the upper and mid dermis, leading to high pressure and hypoxia which causes purpuric papules and hemorrhagic vesicles that erupt into painful ulcers [2, 3]. These ulcers gradually evolve into porcelain-white atrophic scars with punctate telangiectasian known as atrophie blanche, over the course of a few months. Livedoid vasculopathy is known to mainly affect the lower extremities; however, there have also been reports of upper extremity involvement [3, 4]. We describe the first case of livedoid vasculopathy affecting the penis to be reported in the English literature.

## 2. Case

**Figure 1:**
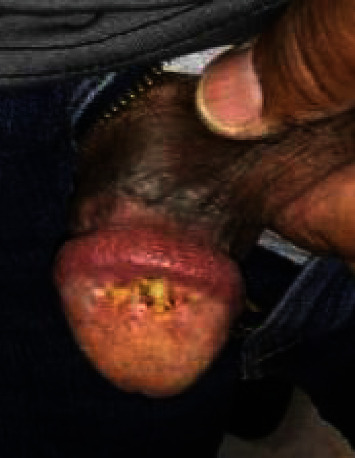
Penile lesion one month after initial presentation.

A 60-year-old male with a history of cerebral artery aneurysm status postclipping several years ago, obesity, prior tobacco use, and hypertension presented to the urology clinic for evaluation of a split urine stream with associated irritation and peeling of the skin of his glans penis which worsened with intercourse (Figure 1). He was prescribed topical clotrimazole and betamethasone and referred to dermatology.

**Figure 2:**
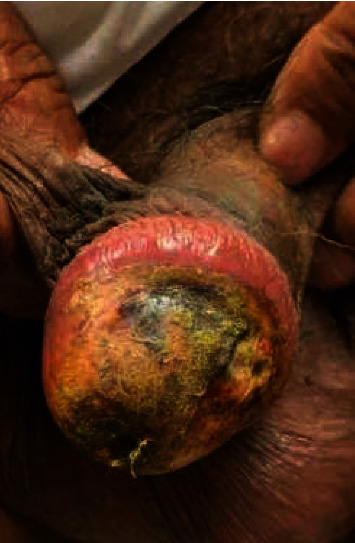
Penile lesion two months after initial presentation.

Three weeks later, at the dermatology clinic, his penile lesion continued to peel, and he was diagnosed with anogenital psoriasis and prescribed triamcinolone. He was followed up in the dermatology clinic after three weeks. By this point, his penile lesion had started ulcerating (Figure 2). He was prescribed arginine for the concern of vascular occlusion as well as petroleum jelly and doxycycline for the concern of infection.

He returned to the urology clinic the next day, and acyclovir was added. He was tested for sexually transmitted infections which were negative. The ulcer was cultured and grew *Streptococcus agalactiae*. Because he did not have any improvement with antibiotics, he was scheduled for a biopsy due to concern of squamous cell carcinoma. A punch biopsy one week later revealed epidermal ulceration with associated parakeratosis, fibrinoid necrosis, and mixed inflammation with mild squamous atypia. The pathologist recommended a repeat biopsy due to the uncertainty of the diagnosis.

At follow-up one week later in the urology clinic, his ulcer had continued to enlarge and the pain had become severe. He was subsequently admitted to the hospital for intravenous antibiotics and further evaluation. He was discharged 2 days later with a prednisone taper and clindamycin.

One week later, he underwent an elliptical biopsy which revealed hyaline thrombi within the lumens of small vessels within the upper to mid dermis with fibrinoid material present in the walls of these vessels as well as the perivascular stroma. Spongiosis was also present with an associated mild perivascular lymphocytic infiltrate with a few scattered neutrophils, but no vasculitis was identified. These findings were most consistent with livedoid vasculopathy. The hypercoagulability workup was negative except for an elevated protein C of 169%.

He was started on 325 mg of aspirin daily and referred to the dermatology clinic, and pentoxifylline 400 mg TID was added to his treatment regimen. He was referred to the vascular medicine clinic one month later and was found to have limited improvement at that visit. He was evaluated in the hematology clinic, and anticoagulation was recommended. In the interim, he was also reevaluated by urology who tried tadalafil without any significant improvement. After a thorough discussion of the risks and benefits of anticoagulation given a history of a clipped cerebral artery aneurysm, the patient agreed to proceed with anticoagulation and was started on apixaban 10 mg BID for one week, followed by 5 mg BID for 6 months.

**Figure 3:**
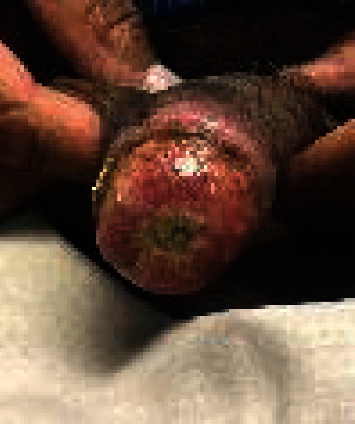
Penile lesion four months after initial presentation, one month after anticoagulation.

**Figure 4:**
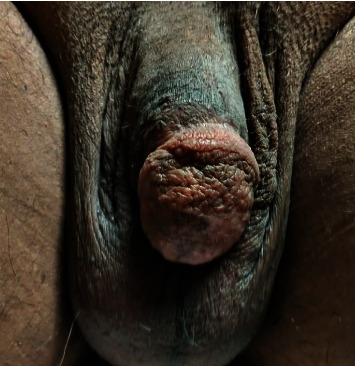
Resolution of penile lesion at 6 months.

He was evaluated every three weeks in the wound care clinic and was found to have significant improvement with the use of collagenase and mupirocin along with continued anticoagulation (Figure 3). Six months after starting anticoagulation and wound care, he followed up in the vascular medicine clinic and was found to have near resolution of his penile ulcer (Figure 4). He was continued on anticoagulation indefinitely with no recurrence at 2 years.

## 3. Discussion

The incidence of livedoid vasculopathy is 1 in 100,000 per year, most commonly between the ages of 15 and 50, with a 3 : 1 female predominance and a characteristic 5-year delay in accurate diagnosis and treatment [5]. Histopathology is the only method of diagnosis. The absence of exuberant neutrophil infiltration, perivascular nuclear fragmentation (leukocytoclasia), and fibrinoid necrosis of blood vessels defines this pathology as a vasculopathy rather than a vasculitis [5].

Although there is currently no standardized treatment, a recent systematic review revealed anticoagulation to be of most benefit, showing improvement in 62 out of 63 patients with livedoid vasculopathy who were started on anticoagulation [6]. Other therapies include danazol, glucocorticoids, and antiplatelets.

Diseases affecting the penile vascular system, both arterial and venous, typically result in erectile dysfunction [7]. Vasculitis may affect the skin of the penis and is almost always associated with systemic vasculitides including granulomatosis with polyangiitis, polyarteritis nodosa, IgA vasculitis, and other forms of vasculitis [8]. Single-organ vasculitis affecting the penis has only been reported in one case [9]. Patients with systemic sclerosis are at risk of developing penile vascular damage in what is known as “sclerodermic penis,” characterized by penile fibrosis [9].

Penile livedoid vasculopathy has not previously been reported in the English literature. Vasculopathies remain a diagnostic challenge for clinicians, which is apparent from our case where the patient needed multiple follow-ups with multiple subspecialists over a protracted period of time before an eventual diagnosis was made. Additionally, a high index of suspicion was required to pursue an invasive diagnostic workup such as getting a biopsy of this sensitive organ. Early diagnosis and treatment are imperative for limiting major morbidity.
